# Moderate Protein Restriction Protects Against Focal Cerebral Ischemia in Mice by Mechanisms Involving Anti-inflammatory and Anti-oxidant Responses

**DOI:** 10.1007/s12035-019-01679-6

**Published:** 2019-07-01

**Authors:** Tayana Silva de Carvalho, Eduardo H. Sanchez-Mendoza, Luiza M. Nascentes, Adriana R. Schultz Moreira, Maryam Sardari, Egor Dzyubenko, Christoph Kleinschnitz, Dirk M. Hermann

**Affiliations:** Department of Neurology, University Hospital Essen, Hufelandstr. 55, 45122 Essen, Germany

**Keywords:** Cerebral metabolism, Ischemic stroke, Middle cerebral artery occlusion, Neuroprotection, Oxidative stress, Protein intake

## Abstract

Food composition influences stroke risk, but its effects on ischemic injury and neurological deficits are poorly examined. While severe reduction of protein content was found to aggravate neurological impairment and brain injury as a consequence of combined energy-protein malnutrition, moderate protein restriction not resulting in energy deprivation was recently suggested to protect against perinatal hypoxia-ischemia. Male C57BL6/j mice were exposed to moderate protein restriction by providing a normocaloric diet containing 8% protein (control: 20% protein) for 7, 14, or 30 days. Intraluminal middle cerebral artery occlusion was then induced. Mice were sacrificed 24 h later. Irrespective of the duration of food modification (that is, 7–30 days), protein restriction reduced neurological impairment of ischemic mice revealed by a global and focal deficit score. Prolonged protein restriction over 30 days also reduced infarct volume, brain edema, and blood-brain barrier permeability and increased the survival of NeuN+ neurons in the core of the stroke (i.e., striatum). Neuroprotection by prolonged protein restriction went along with reduced brain infiltration of CD45+ leukocytes and reduced expression of inducible NO synthase and interleukin-1β. As potential mechanisms, increased levels of the NAD-dependent deacetylase sirtuin-1 and anti-oxidant glutathione peroxidase-3 were noted in ischemic brain tissue. Irrespective of the protein restriction duration, a shift from pro-oxidant oxidative stress markers (NADPH oxidase-4) to anti-oxidant markers (superoxide dismutase-1/2, glutathione peroxidase-3 and catalase) was found in the liver. Moderate protein restriction protects against ischemia in the adult brain. Accordingly, dietary modifications may be efficacious strategies promoting stroke outcome.

**Electronic supplementary material:**

The online version of this article (10.1007/s12035-019-01679-6) contains supplementary material, which is available to authorized users.

## Introduction

Alimentation with protein-rich animal products has greatly fostered human development. At the same time, protein-rich nutrition, specifically with red meat, might elevate health risks, as indicated by a number of population-based studies. Regarding stroke, the Nurses’ Health Study and Health Professionals Follow-Up Study, which prospectively examined 84,010 women and 43,150 men more than 20 years, reported that one serving of red meat per day increases the incidence of stroke events by a factor of 1.13 (95%-confidence interval (CI) 1.04–1.22) [1]. This observation was later confirmed in a meta-analysis on 329,495 subjects followed up over 8–26 years, which found a 1.11 (1.06–1.16)-fold increase of stroke events per red meat serving [2].

The question how food composition influences the severity of ischemic injury and neurological impairment, once a stroke has occurred, has major importance for stroke management. Yet, little data exist on this issue. It is well established that severe malnutrition characterized by low body mass index (BMI) and hypoalbuminemia is associated with poor stroke recovery [3]. How more moderate changes in food intake that are not associated with BMI changes influence ischemic injury is unknown. Randomized studies are lacking on this issue. The diversity of human nutrition habits possibly precludes insights from clinical cohort studies [1, 2].

In rats and gerbils, the delivery of diets containing 0.5–2% protein 3 to 4 weeks in models of global [4–6] or focal [7] cerebral ischemia compromised neurological recovery, increased brain inflammation, increased neuronal injury, and reduced brain plasticity. Yet, such severe protein restriction results in a reduction in the total amount of food ingested, since the animals refuse this chow [4–7]. In such animals, combined protein-energy malnutrition is noted. A single rat study so far examined consequences of a more moderate diet containing 7% protein, which, when administered during pregnancy and lactation to mothers, reduced brain injury but augmented sensorimotor deficits of offspring exposed to unilateral cerebral hypoxia-ischemia at 7 days post-birth [8]. How such moderate protein restriction influences ischemic injury and neurological deficits in the adult brain was unknown. By exposing mice to a normocaloric diet containing 8% protein, which we subsequently exposed to intraluminal middle cerebral artery occlusion (MCAO), we now examined this question. Our hypothesis was that moderate protein restriction reduces neurological deficits and brain injury.

## Materials and Methods

### Legal Issues, Statistical Planning, and Randomization

Experiments were approved by local government authorities (Bezirksregierung Düsseldorf) in accordance with E.U. guidelines (Directive 2010/63/EU) for the care and use of laboratory animals. Sample size calculations determined that 12 animals per group were required for the neurological examinations and histochemical studies, given that the effect size was 1.167, the alpha error was 5%, and the beta error (1–statistical power) was 20%. Experimenters were blinded by a third person not involved in the assessments randomizing the animals, weighing and providing the food pellets. All animals survived the stroke surgery. To minimize the use of laboratory animals, all animals were used both for behavioral studies and histochemical or molecular biological analyses.

### Animal Nutrition and Murinometrics

Thirty-six adult male C57BL6/j mice (8 weeks, 26-30 g; Harlan-Netherlands, Rossdorf, Germany) were randomized to two diets: (a) normal nutrition (C1000; 3518 kcal/kg, 20% (by weight) casein, 13% (by weight) fat; Altromin, Lage, Germany) and (b) protein-reduced nutrition (C1003; 3541 kcal/kg, 8% casein, 13% fat; Altromin). Diets and water were delivered ad libitum over 7, 14, or 30 days. These durations were chosen to obtain a thorough understanding of the animals’ adaptation to the food modification. Animals were then submitted to 30 min intraluminal MCAO. Throughout the study, animals were housed in single cages (Green line IVC Sealsafe PLUS mouse; Tecniplast, Hohenpeißenberg, Germany) in a 12 h/12 h light/dark cycle, in order to quantify food intake. Food consumption and calorie intake were measured daily. Body weight and BMI were measured weekly. Body (i.e., nose–anus) length was determined prior to diet exposure for evaluating BMI (in kg/cm^2^) [9].

### Experimental Procedures

Mice were anesthetized with 1.0–1.5% isoflurane (30% O_2_, remainder N_2_O). Rectal temperature was maintained between 36.5 and 37.0 °C using a feedback-controlled heating system. Cerebral laser Doppler flow (LDF) was recorded using a flexible probe (Perimed, Järfälla, Sweden) attached to the skull overlying the core of the middle cerebral artery territory. A midline neck incision was made. The left common and external carotid arteries were isolated and ligated, and the internal carotid artery was temporarily clipped. A silicon-coated nylon monofilament (0.21 mm tip diameter; Doccol, Sharon, MA, USA) was introduced through a small incision of the common carotid artery and advanced to the circle of Willis for MCAO [10, 11]. Reperfusion was initiated by monofilament removal. Wounds were carefully sutured and anesthesia was discontinued. MCAO was induced during the day cycle of the animals in the surgery facility of the NeuroScienceLab. Behavioral abnormalities and clinical manifestations were checked eight-hourly until 24 h after MCAO. Twenty-four hours later, animals were evaluated using the Clark score [12], which captures general and focal neurological deficits. Immediately before animal sacrifice, plasma samples were obtained after 5 h fasting by cardiac puncture that were used for analysis of total cholesterol, low-density lipoprotein cholesterol (LDL), triglycerides, and glucose levels (ADVIA® 2400; Siemens, Erlangen, Germany). One set of animals (*n* = 12/ group) was transcardially perfused with normal saline followed by 4% paraformaldehyde for histochemical studies. Another set (*n* = 6/ group) was transcardially perfused with normal saline for Western blots and real-time quantitative polymerase chain reaction (qPCR) studies. Brains were cut into 20-μm-thick coronal sections.

### Infarct Volumetry

Coronal sections collected at millimeter intervals across the brain were stained with cresyl violet. Infarct volume was determined by subtracting the area of healthy tissue in the ischemic hemisphere from that in the contralesional hemisphere [11]. Considering that infarct volume was the most rigid stroke readout, infarct volume was defined as primary readout of this study.

### Immunohistochemistry of IgG Extravasation

Brain sections obtained from the rostrocaudal level of the midstriatum were rinsed for 20 min in 0.3% H_2_O_2_ in 70% methanol/ 0.1 M phosphate-buffered saline (PBS), immersed in 0.1 M PBS containing 5% bovine serum albumin (BSA) (05470; Sigma-Aldrich, Darmstadt, Germany), and incubated for 1 h in biotinylated anti-mouse IgG (1:100; Santa Cruz, Heidelberg, Germany), followed by diaminobenzidine tetrahydrochloride (DAB) (D5905; Sigma-Aldrich, Darmstadt, Germany) staining with an avidin-biotin complex peroxidase kit (Vectastain Elite; Vector Labs, Burlingame, CA, U.S.A.) [11]. IgG extravasation was analyzed by evaluating the area covered by IgG in the ischemic brain.

### Terminal Deoxynucleotidyl Transferase-Mediated dUTP Nick End Labeling

Adjacent brain sections were subjected to terminal deoxynucleotidyl transferase-mediated dUTP nick end labeling (TUNEL) using a commercially available In Situ Cell Death Detection kit (Roche, Mannheim, Germany). TUNEL+, that is, DNA-fragmented cells were assessed under an inverted microscope equipped with Apotome optical sectioning (Zeiss Axio Observer.Z1) by counting the total number of labeled cells in the ischemic striatum [11].

### Immunohistochemistry for Neuronal, Microglial, Astrocytic, and Inflammation Markers

Adjacent sections were immersed in 0.1 M PBS containing 0.3% Triton X-100 (PBS-T) and 5% normal donkey serum (D9663; Sigma-Aldrich, Darmstadt, Germany). Sections were incubated overnight at 4 °C in monoclonal rabbit anti-NeuN (1:400; ab177487; Abcam, Cambridge, UK), monoclonal rat anti-CD45 (1:200; 550,539; BD Biosciences, Heidelberg, Germany), polyclonal rabbit anti-ionized calcium binding adaptor protein (Iba)-1 (1:500; Wako Chemicals, Neuss, Germany), monoclonal rat anti-glial fibrillary acidic protein (GFAP) (1:200; 130,300; Invitrogen, Dublin, Ireland), or polyclonal rabbit anti-inducible nitric oxide synthase (iNOS) (1:100; sc-650, Santa Cruz, Heidelberg, Germany) antibodies that were detected with Alexa Fluor-488 or Alexa Fluor-594-labeled secondary antibodies (NeuN, Iba-1, GFAP, iNOS) or biotinylated secondary antibodies followed by DAB staining with an avidin-biotin complex peroxidase kit (Vectastain Elite; Vector Labs, Burlingame, CA, U.S.A.) (CD45). NeuN, Iba-1, GFAP, and iNOS labeling were counterstained with 4′,6-diamidino-2-phenylindole (DAPI) (D9542; Sigma-Aldrich, Darmstadt, Germany). Sections were evaluated under a motorized Zeiss Axio Observer.Z1 inverted epifluorescence microscope equipped with Apotome optical sectioning (NeuN, Iba-1, GFAP, iNOS) or an Olympus X52 microscope (CD45) by counting the total number of NeuN+, CD45+, or iNOS+ cells in the striatum or analyzing the area covered by activated microglia (Iba-1) or reactive astrocytes (GFAP). The latter analysis was preferred to cell countings, since individual cells could not always unequivocally be discriminated. The latter data were shown as percent changes.

### Real-Time Quantitative Polymerase Chain Reaction (qPCR)

From tissue samples harvested from the ischemic middle cerebral artery territory and liver, messenger RNA (mRNA) was extracted using the RNeasy Mini Kit (Qiagen, Hilden, Germany). mRNA was converted to cDNA using the high-capacity RNA-to-cDNA kit (Thermo Fisher Scientific, Waltham, MA, U.S.A). Real-time qPCR was performed in a StepOnePlus real-time PCR instrument (Thermo Fisher Scientific, Waltham, MA, USA) using primers designed or selected by the PubMed primer BLAST tool (https://blast.ncbi.nlm.nih.gov/) (Suppl. Table 1). Melting curves were used to confirm the efficiency of the primers. β-glucuronidase (β-Gluc) was used as housekeeping gene; brain and liver tissue from healthy mice served as control. Data were finally normalized that animals on normal diet were set as 1. Results were quantified using the 2^−∆∆Ct^ method [13]. PCR was performed in triplicates, of which mean values were computed for each animals.

### Western Blots

During mRNA extraction, protein samples were collected after bromocholoropropane (B9673; Sigma-Aldrich, Darmstadt, Germany) separation. Ethanol was added and samples centrifuged at 12,000×*g* for 5 min. This procedure was repeated twice. The resulting pellet was suspended in 4% sodium dodecyl sulfate (SDS) (436,143; Sigma-Aldrich, Darmstadt, Germany). Protein content was measured using the Bradford method. Equal amounts of protein (20 μg) were loaded on 10% SDS-polyacrylamide gels, submitted to SDS-polyacrylamide gel electrophoresis (PAGE), and transferred onto polyvinylidene fluoride (PVDF) membranes (Bio-Rad, Hercules, CA, USA). Membranes were blocked by 5% nonfat-dried milk (M7409; Sigma-Aldrich, Darmstadt, Germany) in 50 mM Tris-buffered saline (TBS) containing 0.1% Tween (P9416; Sigma-Aldrich, Darmstadt, Germany) for 1 h at room temperature, washed and incubated overnight at 4 °C with monoclonal rabbit anti-sirtuin-1 (Sirt-1; 1:2000; ab32441; Abcam, Cambridge, UK), polyclonal rabbit anti-glutathione peroxidase-3 (Gpx-3; 1:2000; ab59524; Abcam, Cambridge, UK), and polyclonal rabbit anti-β-actin (1:10000; 4967; Cell Signaling, Frankfurt, Germany) antibody. The next day, membranes were washed and incubated with secondary donkey anti-rabbit antibody. Blots were revealed using a chemiluminescence kit and scanned using amyECL Imager (Thermo Fisher Scientific, Waltham, MA, U.S.A.). Sirt-1 and Gpx-3 abundance was densitometrically evaluated in three independent experiments. The relative abundance of Sirt-1 and Gpx-3 was normalized to protein loading as determined in β-actin blots.

### Statistics

Statistical analyses were performed using SPSS for Windows. Murinometric data, nutritional data, and LDF recordings were analyzed by repeated-measurement ANOVA followed by unpaired *t* tests as post hoc tests. Neurological deficits, histochemical data, Western blots, and real-time qPCR data were analyzed by *t* tests. To explore the relationship of protein and calorie intake with infarct volume and neurological deficits, two-tailed Pearson’s correlations were computed. LDF recordings, murinometric, nutritional, and real-time qPCR data are presented as mean ± S.D. values. Neurological deficits, histochemical data, and Western blots are shown as median ± interquartile range box-blots with minimum/maximum data as whiskers. *P* values < 0.05 were defined to indicate statistical significance.

## Results

### Moderate Protein Restriction Does Not Alter Major Murinometric and Nutritional Variables but Reduces Plasma LDL and Triglycerides

Murinometric assessments did not display any changes in body weight (Suppl. Fig. 1A-C) or BMI (Suppl. Fig. 1D-F) over up to 30 days in mice exposed to protein restriction compared with mice receiving normal diet. Likewise, the total amount of food ingested (Suppl. Fig. 1G-I) and calorie intake (Suppl. Fig. 1J-L) prior to MCAO did not differ between groups. After MCAO, until animal sacrifice, animals receiving the protein-reduced diet exhibited higher food (Suppl. Fig. 1G-I) and calorie (Suppl. Fig. 1J-L) intake than animals receiving the normal diet. This stabilized food and calorie intake resembles observations previously made in ischemic mice exposed to intermittent fasting prior to MCAO [14]. Food and calorie intake post-MCAO in animals on protein restriction was very similar to pre-stroke food and calorie intake. This was not the case in animals on normal diet. In mice exposed to 30, but not 7 or 14 days protein restriction, plasma cholesterol [158.3 ± 19.5 vs. 252.1 ± 120.0 mg/dl, *p* < 0.05], LDL [13.6 ± 14.1 vs. 32.7 ± 29.7 mg/dl, *p* < 0.05], and triglyceride [177.1 ± 74.6 vs. 259.3 ± 147.2 mg/dl, *p* < 0.05] levels were significantly lower than in mice on normal diet (Suppl. Table 2). Glucose levels did not differ between groups (Suppl. Table 2). The appearance, color, and size of stool samples did not differ between groups (not shown). Behavioral abnormalities (e.g., hypoactivity) or fur changes were absent in mice exposed to protein restriction.

### Protein Restriction Decreases Neurological Deficits, Ischemic Injury, Brain Edema, and Blood-Brain Barrier Permeability

**Fig. 1:**
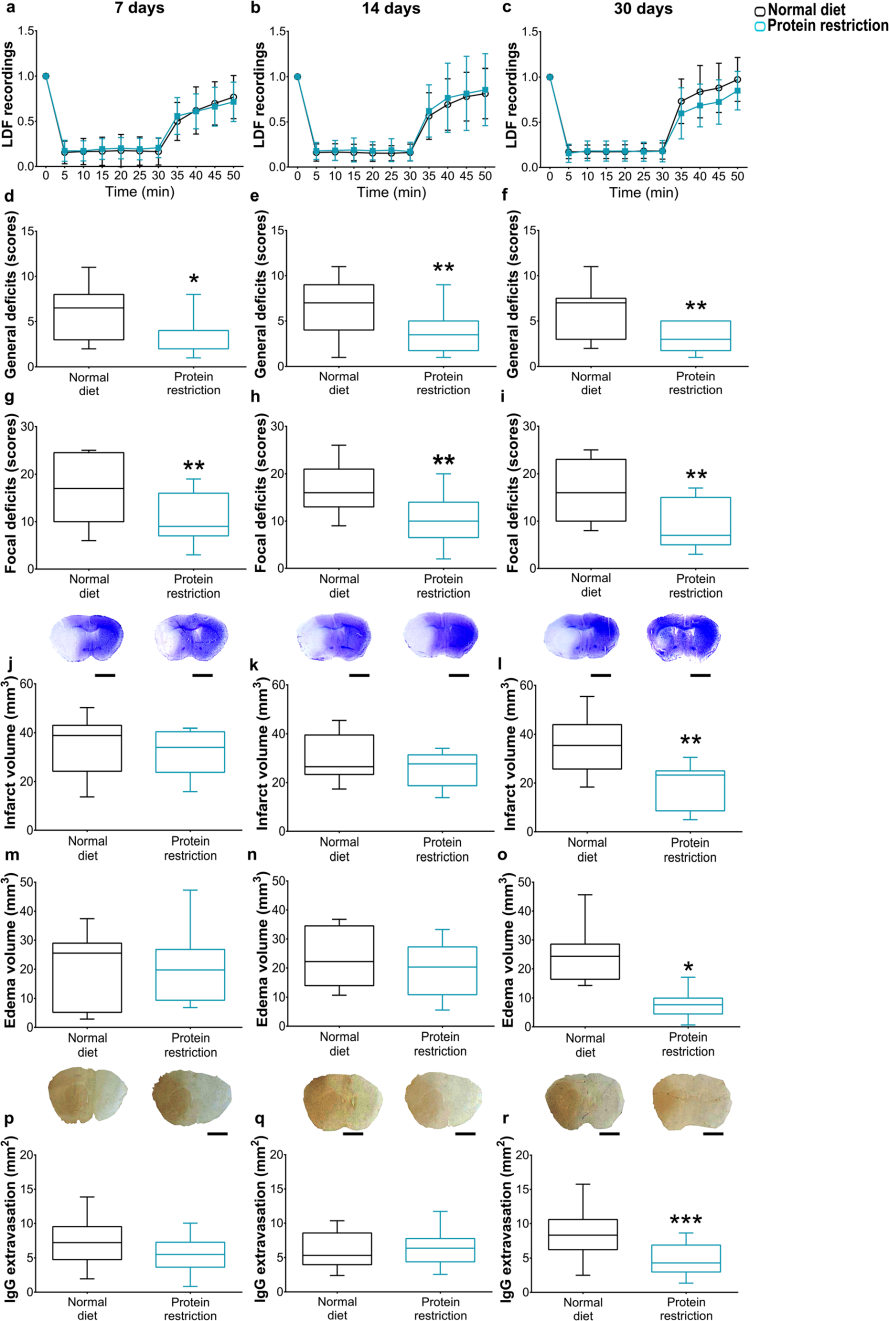
Protein restriction decreases neurological deficits, infarct volume, brain edema, and blood-brain barrier permeability. **a**–**c** Laser Doppler flow (LDF) recordings above the core of the middle cerebral artery territory, **d**–**f** general neurological deficits evaluated by the Clark score, **g**–**i** focal neurological deficits examined by the Clark score, **j**–**l** infarct volume and **m**–**o** edema volume outlined on cresyl violet-stained brain sections, and (**p**–**r**) blood-brain barrier permeability in the striatum assessed by IgG extravasation analysis in mice exposed to normal or protein-reduced diet for 7 days (**a**, **d**, **g**, **j**, **m**, **p**), 14 days (**b**, **e**, **h**, **k**, **n**, **q**), or 30 days (**c**, **f**, **i**, **l**, **o**, **r**), followed by 30-min intraluminal MCAO and 24 h reperfusion. Representative photographs are shown. Data are presented as mean ± S.D. values (**a**–**c**) or median ± interquartile range box-blots with minimum/maximum data as whiskers (**d**–**r**). Bars in (**e**, **f**, **g**, **h**, **i**, **l**), 1 mm. ***p < 0.001; **p < 0.01; *p < 0.05 compared with corresponding normal diet (*n* = 12 animals/group)

**Fig. 2:**
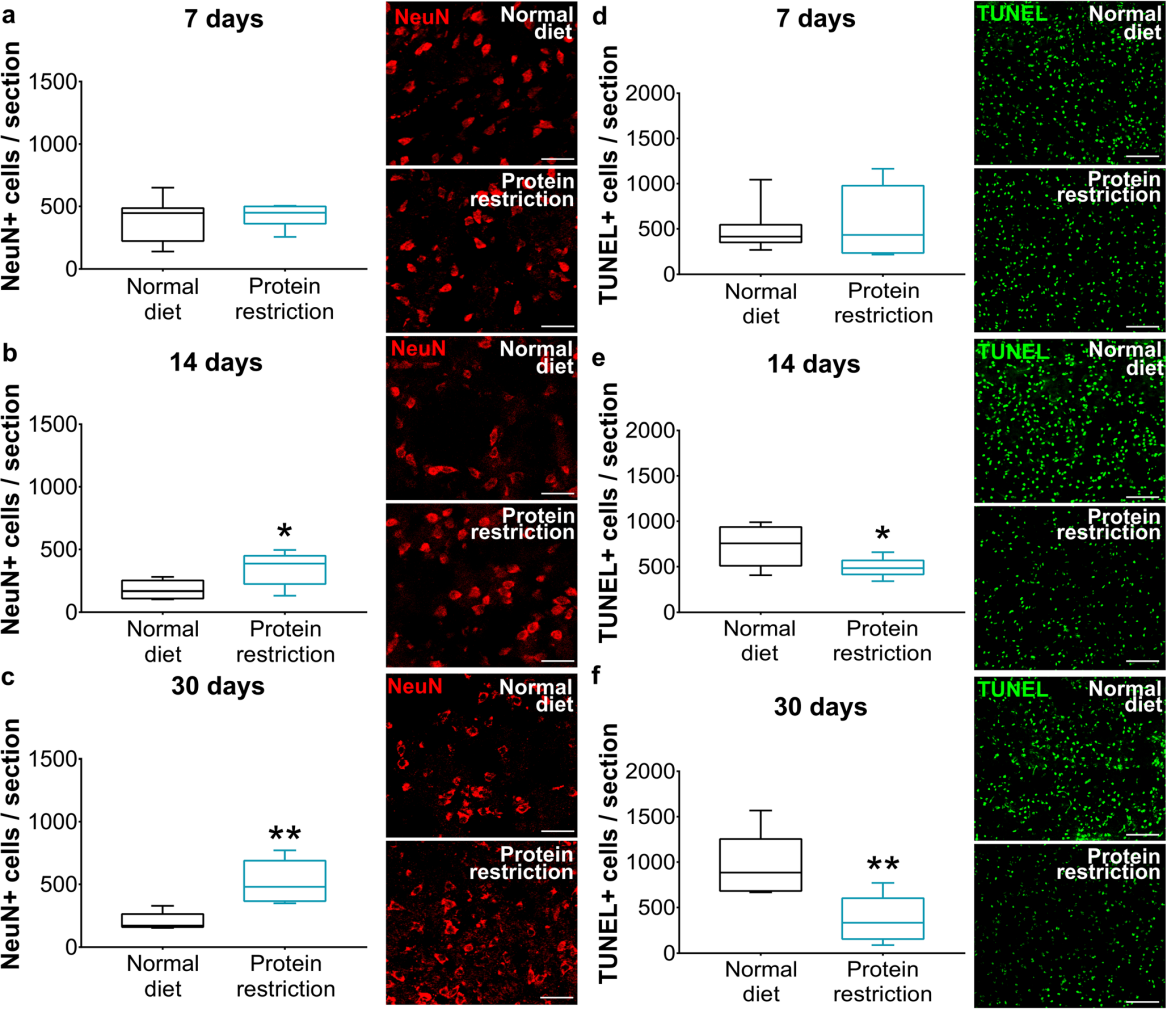
Protein restriction promotes post-ischemic neuronal survival. **a**–**c** Number of NeuN+ surviving neurons and **d**–**f** number of DNA-fragmented, that is, irreversibly injured, TUNEL+ cells in the ischemic striatum of mice exposed to normal or protein-reduced diet over 7 days (**a**, **d**), 14 days (**b**, **e**), or 30 days (**c**, **f**), followed by 30-min intraluminal MCAO and 24 h reperfusion. Representative microphotographs are shown. Data are median ± interquartile range box-blots with minimum/ maximum data as whiskers. Bars, 100 μm. **p < 0.01; *p < 0.05 compared with normal diet (*n* = 12 animals/group)

Cerebral LDF recordings during and after MCAO did not differ between groups. LDF reproducibly decreased to ~ 15–20% of baseline values during MCAO, followed by the return of LDF to baseline values within 20 min after reperfusion (Fig. 1a–c). Irrespective of the duration of food modification (7, 14, or 30 days), general (Fig. 1d–f) and focal (Fig. 1g–i) neurological deficits after 24 h were significantly reduced by protein restriction. Conversely, infarct volume (Fig. 1j–l), brain edema (Fig. 1m–o), and blood-brain barrier permeability assessed by serum IgG extravasation (Fig. 1p–r) was significantly decreased by protein restriction when administered over 30, but not 7 or 14 days. The number of surviving NeuN+ neurons in the ischemic striatum was significantly increased (Fig. 2a–c), whereas the number of irreversibly injured, that is, TUNEL+ cells was significantly reduced (Fig. 2d–f) by 14 days and more pronounced 30 days protein restriction.

To further evaluate links between daily protein intake with infarct volume and neurological deficits, Pearson’s correlations were calculated. These Pearson’s correlations revealed that after 30 days [r = 0.387, *p* = 0.001], but not 7 days [r = 0.004, *p* = 0.350] or 14 days [r = 0.071, *p* = 0.206] of protein restriction, protein intake was positively correlated with infarct volume (Suppl. Fig. 2). Independent of the duration of protein restriction (7, 14, or 30 days), protein intake was positively correlated with general and focal neurological deficits (Suppl. Fig. 2).

### Protein Restriction Differentially Influences Brain Leukocyte Infiltration and Microglial Activation

**Fig. 3:**
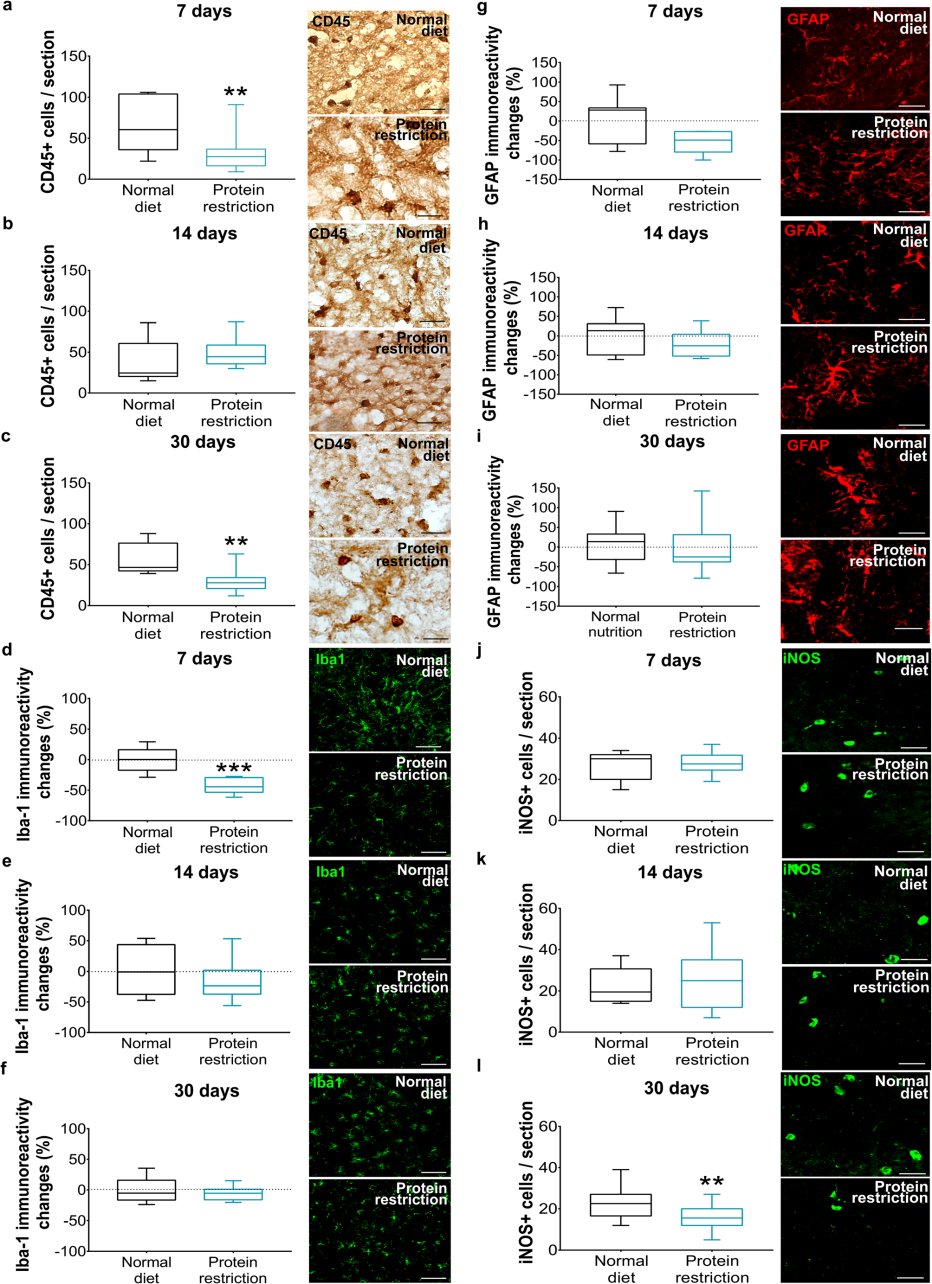
Protein restriction differentially influences brain leukocyte infiltration, microglial activation, and iNOS formation. **a**–**c** Number of CD45+ leukocytes, **d**–**f** immunoreactivity for microglia marker Iba-1, **g**–**i** immunoreactivity for astrocytic marker GFAP, and **j**–**l** number of iNOS+ cells in the ischemic striatum of mice exposed to normal or protein-reduced diet for 7 days (**a**, **d**, **g**, **j**), 14 days (**b**, **e**, **h**, **k**), or 30 days (**c**, **f**, **i**, **l**), followed by 30 min intraluminal MCAO and 24 h reperfusion. Representative microphotographs are shown. Data are median ± interquartile range box-blots with minimum/maximum data as whiskers. Bars, 100 μm. **p < 0.01; *p < 0.05 compared with normal diet (*n* = 12 animals/group)

Protein restriction significantly decreased the invasion of CD45+ leukocytes into ischemic brain tissue, when imposed to 7 or 30 days, but not 14 days (Fig. 3a–c). On the other hand, microglial activation, evaluated by Iba-1 immunohistochemistry, was significantly reduced by 7 days, but not 14 or 30 days protein restriction (Fig. 3d–f). Astrogliosis, examined by GFAP immunohistochemistry, was not influenced by protein restriction (Fig. 3g–i). The number of cells immunopositive for iNOS, a marker of pro-inflammatory M1 macrophages/microglial cells [15], was significantly reduced by protein restriction, when imposed for 30 days, but not 7 or 14 days (Fig. 3j–l). Based on their size and shape, the iNOS+ cells had the appearance of microglial cells.

### Protein Restriction Upregulates the NAD-Dependent Deacetylase Sirtuin-1, Downregulates Interleukin-1β, and Upregulates Glutathione Peroxidase-3

**Fig. 4:**
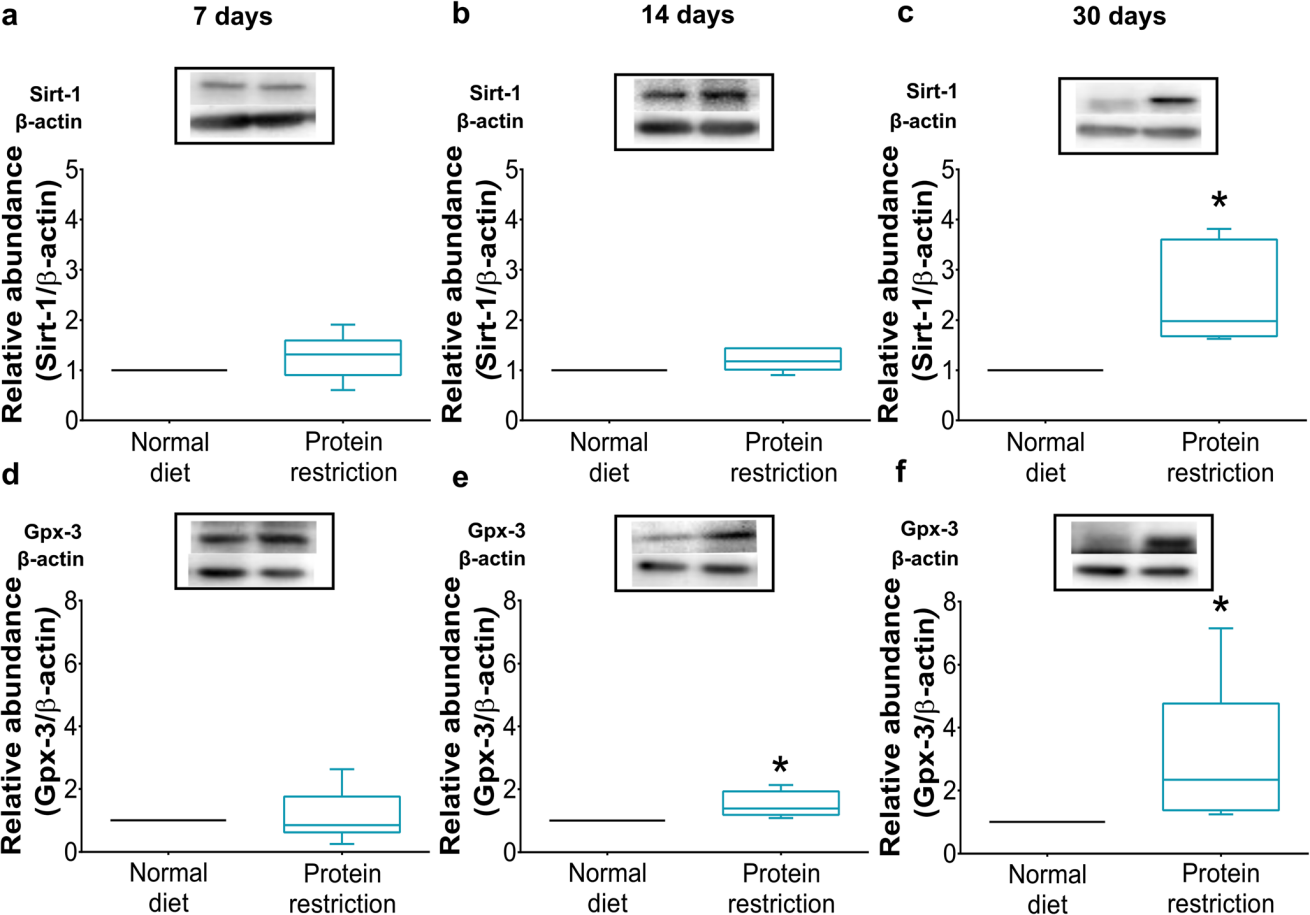
Protein restriction increases sirtuin-1 and glutathione peroxidase-3 abundance in ischemic brain tissue. Western blot analysis of **a**–**c** sirtuin-1 (Sirt-1) and **d**–**f** glutathione peroxidase-3 (Gpx-3) protein in ischemic brain tissue of mice exposed to normal or protein-reduced diet for **a**, **d** 7 days, **b**, **e** 14 days, or **c**, **f** 30 days, followed by 30 min intraluminal MCAO and 24 h reperfusion. Representative Western blots are also shown. Data are median ± interquartile range box-blots with minimum/ maximum data as whiskers. *p < 0.05 compared with normal diet (*n* = 6 animals/ group)

Real-time qPCR showed that protein restriction did not alter the expression of metabolism-related, pro-inflammatory, and anti-oxidant genes in ischemic brain tissue, when imposed for 7 or 14 days, but increased the level of *sirtuin-1* (*Sirt-1*) mRNA, which encodes a NAD-dependent deacetylase that stabilizes mitochondrial function and metabolism partly by deacetylating the transcription regulator peroxisome proliferator-activated receptor-γ coactivator-1α (PGC-1α) [15]; reduced the level of *interleukin-1β* (*Il-1β*) mRNA, which encodes a pro-inflammatory cytokine strongly expressed by M1 microglial cells [15]; and increased the level of *glutathione peroxidase-3* (*Gpx-3*) mRNA, which encodes an anti-oxidant enzyme that degrades hydrogen peroxide [16, 17], in ischemic brain tissue, when imposed for 30 days (Table 1). Western blots revealed that the abundance of Sirt-1 and Gpx-3 proteins was increased after protein restriction over 30 days and in case of Gpx-3 less pronounced also over 14 days (Fig. 4). The metabolic markers *insulin-like growth factor-1* (*Igf-1*) mRNA, which encodes a growth factor with insulin-like properties; *insulin receptor* (*Insr*) mRNA; and *glucose transporter-1* (*Glut-1*) mRNA [18] were not influenced by protein restriction, as was *nuclear factor*-*κb* (*Nf*-*κb*) mRNA, which encodes a transcription factor deacetylated by Sirt-1 that controls *Il-1β* expression [19], or *superoxide dismutase-1* (*Sod-1*) mRNA, which encodes a dismutase degrading superoxide anions to hydrogen peroxide [16] (Table 1).

**Table 1:** Responses of metabolism-related, inflammatory, and anti-oxidant genes in the ischemic brain of mice exposed to protein restriction

Brain	*Sirt-1*	*Igf-1*	*Insr*	*Glut-1*	*Il-1β*	*Nf-κb*	*Sod-1*	*Gpx-3*
7 days								
Normal diet	1.13 ± 0.60	3.60 ± 3.26	1.52 ± 0.74	1.18 ± 0.44	5.40 ± 3.61	1.55 ± 0.60	0.90 ± 0.34	0.13 ± 0.11
Protein restriction	1.11 ± 0.40	2.30 ± 1.65	1.14 ± 0.44	1.05 ± 0.42	4.12 ± 3.70	1.05 ± 0.40	0.80 ± 0.31	0.09 ± 0.01
14 days								
Normal diet	0.60 ± 0.23	1.64 ± 1.33	1.32 ± 1.11	1.17 ± 0.44	3.50 ± 1.60	3.00 ± 1.23	1.01 ± 0.34	0.30 ± 0.15
Protein restriction	0.50 ± 0.20	1.83 ± 1.22	0.80 ± 0.30	0.90 ± 0.55	3.16 ± 1.95	2.25 ± 0.73	1.12 ± 0.46	0.30 ± 0.42
30 days								
Normal diet	0.30 ± 0.11	1.84 ± 1.12	0.70 ± 0.30	1.14 ± 0.63	5.72 ± 3.20	2.35 ± 1.65	0.70 ± 0.30	0.40 ± 0.47
Protein restriction	0.60 ± 0.20*	0.83 ± 0.83	0.63 ± 0.20	0.90 ± 0.24	1.64 ± 0.92*	1.74 ± 0.66	0.63 ± 0.14	5.17 ± 4.00*

Data are real-time quantitative polymerase chain reaction (qPCR) results, expressed as mean ± SD values

*p < 0.05 compared with normal diet (*n* = 6 animals/group; analyzed in triplicates, for which mean values were formed)

### Protein Restriction Regulates Metabolism-Related, Pro-Oxidant and Anti-Oxidant Genes in the Liver

Real-time qPCR showed that protein restriction regulated genes encoding metabolism-related genes, pro-oxidant and anti-oxidant enzymes in the liver within 7 days. Thus, *Sirt-1* mRNA was upregulated, *NADPH oxidase-4* (*Nox-4*) mRNA, which encodes a protein that catalyzes the production of superoxide free radicals by transferring electrons to oxygen from NADP [20], was downregulated, and *superoxide dismutase-2* (*Sod-2*) mRNA and *catalase* (*Cat*) mRNA, which encodes another peroxidase [17], were upregulated by protein restriction (Table 2). After 14-day exposure to protein-reduced diet, liver levels of *Sirt-1* mRNA, *Nox-4* mRNA, and *Cat* mRNA were reversed to levels in mice receiving normal diet, and liver levels of *Sod-1* mRNA, *Sod-2* mRNA, and *Gpx-3* mRNA were increased (Table 2). Prolonged 30-day protein restriction reregulated metabolism-related and anti-oxidant markers. Thus, *Sod-1* mRNA and *Sod-2* mRNA were restored to levels in normal diet mice, and *Sirt-1* mRNA, *Igf-1* mRNA, and *Gpx-3* mRNA were elevated (Table 2).

**Table 2:** Responses of metabolism-related, inflammatory, pro- and anti-oxidant genes in the liver of mice exposed to protein restriction

Liver	*Sirt-1*	*Igf-1*	*Insr*	*Glut-2*	*Nf-κb*	*Nox-4*	*Sod-1*	*Sod-2*	*Gpx-3*	*Cat*
7 days										
Normal diet	0.75 ± 0.20	0.75 ± 0.43	0.40 ± 0.48	0.90 ± 0.48	1.66 ± 0.87	1.08 ± 0.49	0.86 ± 0.23	1.00 ± 0.35	0.89 ± 0.27	0.39 ± 0.18
Protein restriction	1.60 ± 0.80*	0.80 ± 0.75	0.34 ± 0.40	0.99 ± 0.70	1.40 ± 0.93	0.52 ± 0.20*	0.99 ± 0.53	2.05 ± 0.88*	0.97 ± 0.44	2.24 ± 1.40*
14 days										
Normal diet	0.30 ± 0.37	0.60 ± 0.37	0.42 ± 0.40	0.88 ± 0.48	0.75 ± 0.56	0.86 ± 0.35	0.98 ± 0.30	0.99 ± 0.14	2.22 ± 2.06	0.44 ± 0.48
Protein restriction	0.30 ± 0.14	0.76 ± 0.64	0.24 ± 0.40	1.26 ± 1.06	0.68 ± 0.37	0.67 ± 0.21	2.29 ± 1.33*	2.73 ± 1.60*	6.00 ± 3.50*	0.45 ± 0.63
30 days										
Normal diet	0.80 ± 0.18	0.22 ± 0.31	0.24 ± 0.26	1.20 ± 0.31	0.67 ± 0.28	1.17 ± 0.31	1.03 ± 0.08	1.75 ± 1.66	0.76 ± 0.31	0.69 ± 0.54
Protein restriction	1.50 ± 0.64*	1.20 ± 0.40**	0.70 ± 0.63	1.21 ± 0.95	0.74 ± 0.70	1.26 ± 0.34	0.78 ± 0.40	1.60 ± 0.89	1.70 ± 0.71*	0.98 ± 0.97

Data are real-time quantitative polymerase chain reaction (qPCR) results, expressed as mean ± SD values

*p < 0.05/**p < 0.01 compared with normal diet (*n* = 6 animals/group; analyzed in triplicates, for which mean values were formed)

## Discussion

By exposing adult mice to intraluminal MCAO that had been submitted to a protein-reduced diet for 7, 14, or 30 days, we show that protein restriction protects against focal cerebral ischemia. Irrespective of the duration of food modification (7–30 days), post-ischemic neurological deficits were reduced by protein restriction. In contrast to these behavioral improvements, only prolonged protein restriction over 30 days reduced infarct volume, brain edema, and blood-brain barrier permeability. Neuroprotection by protein restriction went along with increased neuronal survival in the ischemic striatum, reduced brain infiltration of CD45+ leukocytes, and reduced the expression of iNOS and interleukin-1β. As potential mechanism, increased expression of the NAD-dependent deacetylase Sirt-1 and increased expression of anti-oxidant Gpx-3 were noted in ischemic brain tissue. Robust responses of oxidative stress markers, indicating a shift from pro-oxidant (Nox-4) to anti-oxidant (Sod-1, Sod-2, Gpx-3, Cat) enzymes, were detected in the liver.

Previous studies in rat and gerbil models of global and focal cerebral ischemia found that protein restriction compromises neurological recovery in motor-coordination tests [5, 7], increases brain inflammation via activation of NF-κb [4], increases neuronal injury [5], and decreases neuronal plasticity, evaluated by the axonal and synaptic proteins growth-associated protein-32, synaptophysin, and synaptosomal-associated protein-25 [6]. These studies have in common that far more severe protein restriction (0.5–2% protein, in all studies casein) was imposed for 3 to 4 weeks. Such severe protein restriction results in a reduction in the total amount of food ingested, since the animals refuse this chow [4–7]. Combined protein-energy malnutrition with a loss of body weight was noted in these animals. A single study so far examined consequences of a more moderate diet containing 7% protein (soybean protein), which, when administered during pregnancy and lactation to mothers, reduced brain injury but augmented sensorimotor deficits and impaired homing behavior of offspring exposed to unilateral cerebral hypoxia-ischemia at 7 days post-birth [8]. The improvement of ischemic injury by protein restriction in this former study goes in line with our study. Unlike this earlier study, we observed an improvement of neurological deficits evaluated with a global and focal behavioral score in adult mice exposed to focal cerebral ischemia. Differences of animal age (adult vs. newborn), ischemia models (intraluminal MCAO vs. unilateral hypoxia-ischemia), diets (casein or soybean protein as protein source), or species (mice vs. rats) may explain the different findings of this previous study [8] and the present one. In this previous study, animals also developed body weight loss [8]. Interestingly, our findings resemble observations following short-term dietary restriction by exposure to a protein-free diet or complete fasting for 6 days in rat models of intraluminal or peripheral MCAO, where similar to the present study reduced infarct volume and decreased motor-coordination deficits were found [21]. Unlike in the present study, body weight was again significantly reduced by both diet protocols [21]. That in our study protein restriction induced neuroprotection without provoking body weight loss is noteworthy. It indicates that, unlike hypothesized earlier [22], body weight loss is not a necessity for neuroprotection to occur subsequent to dietary restriction.

As potential mechanism, protein restriction in the present study reduced plasma cholesterol, LDL, and triglyceride levels and decreased the infiltration of CD45+ leukocytes into the ischemic brain. At the time-point examined (24 h post-MCAO), leukocyte infiltrates are predominated by polymorphonuclear neutrophil (PMN) granulocytes. Using strategies of antibody-mediated PMN depletion or prevention of PMN brain entry, we have previously shown that PMN contribute to ischemic injury after intraluminal MCAO [23] and that PMN furthermore mediate injury-aggravating effects of hypercholesterolemia induced by a lipid-rich Western diet [24]. In the present study, the reduced brain leukocyte infiltration after protein restriction was associated with decreased iNOS and *Il-1β* mRNA levels, which are strongly expressed by pro-inflammatory M1 microglial cells in the ischemic brain [15]. In our study, the iNOS and *Il-1β* mRNA responses dissociated from patterns of microglial activation, which was reduced by 7 days, but not 14 or 30 days protein restriction. It is conceivable that microglial differentiation shifted towards a neuroprotective M2 phenotype upon protein restriction. The expression of iNOS and Il-1β under inflammatory conditions is tightly controlled by the transcription factor *Nf*-*κb* [19], which, as we further showed, was not regulated by protein restriction on the mRNA level.

Neuroprotection by protein restriction was associated with increased expression of the NAD-dependent deacetylase Sirt-1 (both on the mRNA and protein level) and increased expression of anti-oxidant *Gpx-3* mRNA in ischemic brain tissue. Independent of the duration of food modification, robust responses of oxidative stress markers (downregulation of pro-oxidant *Nox-4*, upregulation of *Sod-1/2*, *Gpx-3*, and/or *Cat* mRNAs) were also found in the liver. Sirt-1 has a large variety of actions in the healthy and injured brain, stabilizing cellular energy metabolism partly by deacetylating PGC-1α [19], which, besides others, results in the acquisition of ischemic tolerance. Via different downstream targets, mitochondrial energy coupling is promoted, reactive oxygen species formation reduced, and anti-oxidant capacity increased. Our data suggest that Sirt-1 played a role in the regulation of the above pro- and anti-oxidant enzymes. Further studies will be required to elucidate whether elevated Sirt-1 causally contributed to neuroprotection by protein restriction. Interestingly, *Sirt-1*^*−/−*^ was previously found to exacerbate ischemic injury in mice exposed to intraluminal MCAO but failed to abolish protective effects of calorie restriction [25]. The here presented study complements studies on calorie restriction [26], showing that changes in food composition may similarly induce neuroprotection as changes in the total food or calorie amount. In both types of diet modification, anti-inflammatory and anti-oxidant responses seem to be instrumental for the promotion of stroke outcome.

In view of the important role of food composition for stroke management, several questions still remain to be explored, namely (a) effects of diet modifications initiated after focal cerebral ischemia on neurological recovery and brain remodeling, (b) effects of different protein origins (mammalian, poultry vs vegetarian sources) on ischemic injury and neurological recovery, and (c) effects of diet composition depending on nutrition status and the presence of life style-related risk factors (e.g., hyperlipidemia, diabetes). We need to be aware that nutrition and digestion cannot be transferred one-to-one from rodents to human patients. If thoughtfully addressed, rodent studies might allow us to deduct working hypotheses how modifications in food composition could be used for alleviating stroke consequences.

## Electronic Supplementary Material

ESM 1 (PDF 650 kb)
