# Supplementary material for: Moderate Protein Restriction Protects Against Focal Cerebral Ischemia in Mice by Mechanisms Involving Anti-inflammatory and Anti-oxidant Responses
Source: Mol Neurobiol. 2019 Jul 1;56(12):8477–88. doi: 10.1007/s12035-019-01679-6 (PMC6835038; doi:10.1007/s12035-019-01679-6)
Supplement: Supplementary file 1 — (PDF 650 kb) [file 12035_2019_1679_MOESM1_ESM.pdf]

Moderate protein restriction protects against focal cerebral ischemia in mice by mechanisms involving anti-inflammatory and anti-oxidant responses

[1]Tayana Silva de Carvalho, MSc; [1]Eduardo H. Sanchez-Mendoza, PhD; [1]Luiza M. Nascentes Melo, MSc; [1]Adriana R. Schultz Moreira, PhD; [1]Maryam Sardari, PhD; [1]Egor Dzyubenko, PhD; [1]Christoph Kleinschnitz, MD; [1]Dirk M. Hermann, MD

Department of Neurology, University Hospital Essen, Essen, Germany

Running title: Protein restriction protects against focal cerebral ischemia

Correspondence: Prof. Dirk M. Hermann, MD

Department of Neurology, University Hospital Essen

Hufelandstr. 55, D-45122 Essen, Germany

Phone: +49-201-723-2814, Fax: +49-201-723-5534

E-mail: dirk.hermann@uk-essen.de

ORCID: https://orcid.org/0000-0003-0198-3152

Supplemental Figures and Tables:

Supplemental Figures:

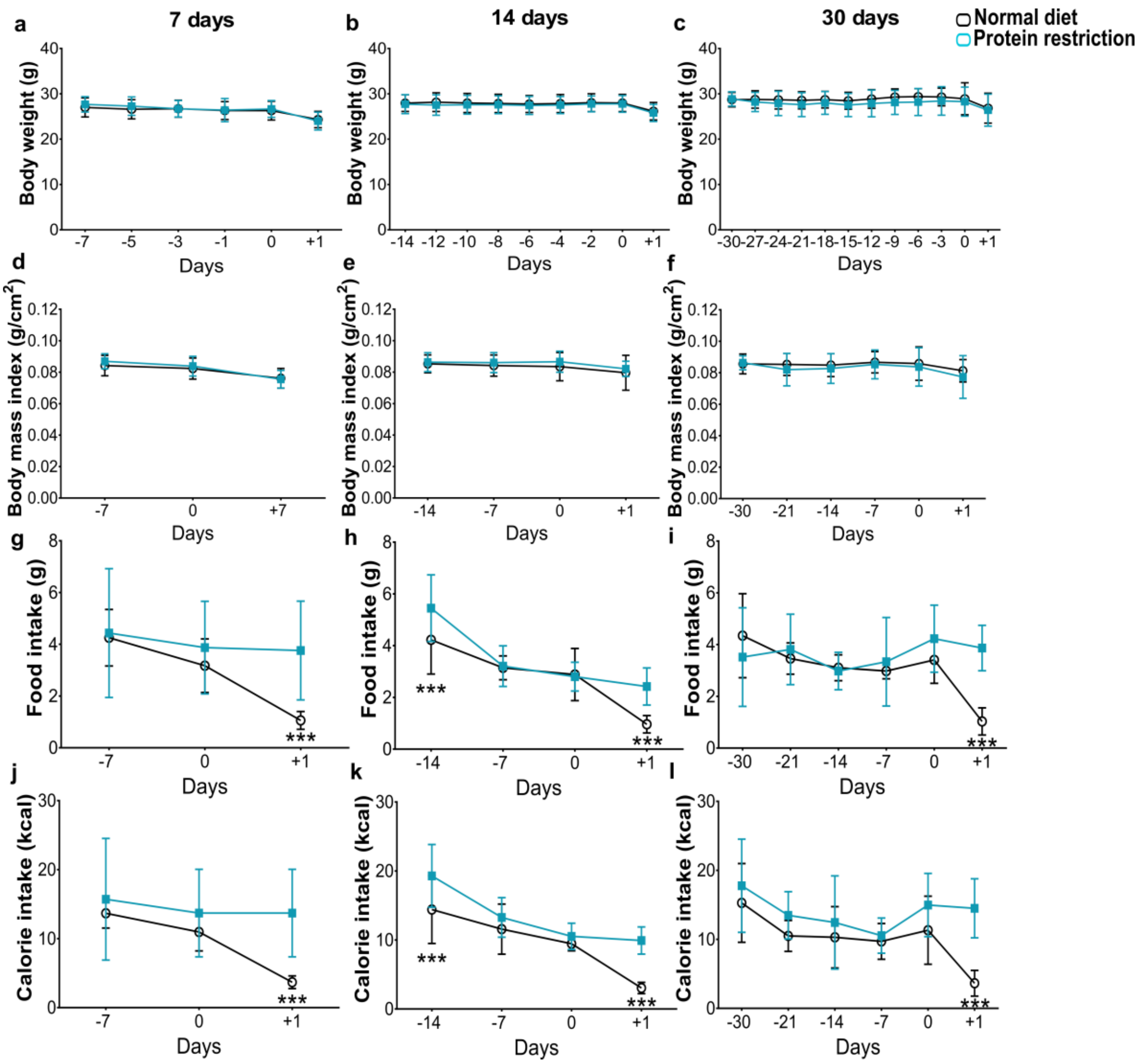

Suppl. Figure 1. Protein restriction does not alter major murinometric and nutritional variables prior to ischemia. (a-c) Body weight, **(d-f)** body-mass index (BMI), **(g-i)** daily food intake, **(j-l)** daily calorie intake in mice receiving normal or protein-reduced diet for 7 days **(a, d, g, j)**, 14 days **(b, e, h, k)** or 30 days **(c, f, i, l)**, followed by 30 minutes intraluminal MCAO and 24 hours reperfusion. The x-axis depicts the time-point with respect to MCAO (=day 0). Note the elevated food (**g-i**) and calorie (**j-l**) intake during the 24 hours after MCAO (day +1) in animals receiving protein-reduced compared with normal diet. Hence, food and calorie intake post-MCAO in mice exposed to protein restriction was very similar to pre-stroke food and calorie intake. ***p<0.001 for protein restriction compared with normal diet (n=12 animals/ group).

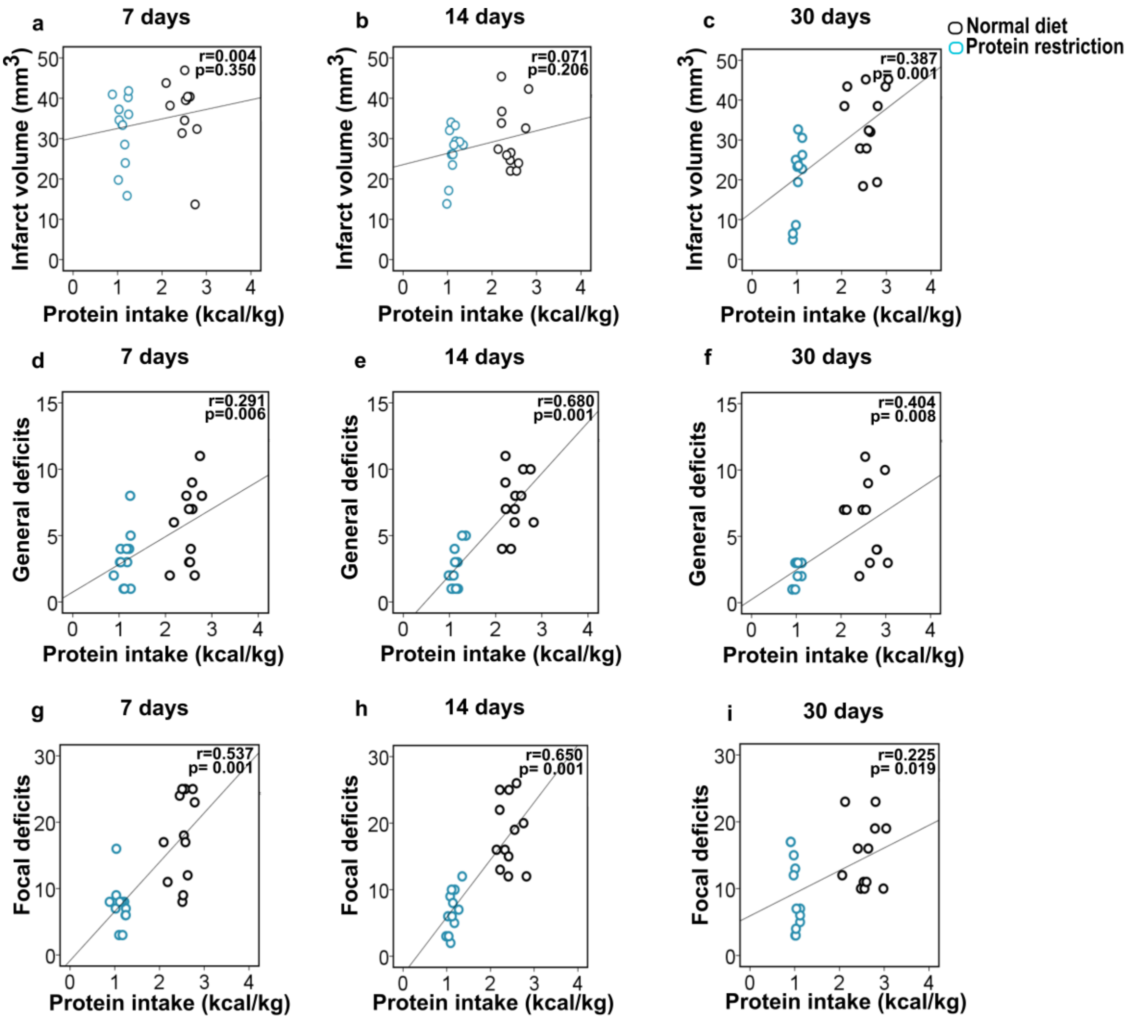

Suppl. Figure 2. Correlation of daily protein intake with infarct volume and neurological deficits. Pearson's correlations of daily protein intake with **(a-c)** infarct volume, **(d-f)** general neurological deficits and **(g-i)** focal neurological deficits in animals exposed to normal or protein-reduced diet for 7 days **(a, d, g)**, 14 days **(b, e, h)** or 30 days **(c, f, i)**, followed by 30 minutes intraluminal MCAO and 24 hours reperfusion. Correlation coefficients (r), p values and univariate regression lines are shown. Note the positive correlation of protein intake with infarct volume in mice exposed to food manipulation for 30 days, but not 7 or 14 days. Protein intake was positively correlated with general and focal neurological deficits in all three experimental conditions (food manipulation for 7, 14 and 30 days).

Supplemental Tables:

Supplemental Table 1. List of PCR primers.

Primer	Sequence (5'->3')		Tm	G-C (%)	Gene bank number
Sirt-1	Forward	GATGACAGAACGTCACACGC	59.56	55.00	NM_019812.3
	Reverse	ATTGTTCGAGGATCGGTGCC	60.46	55.00	
Igf-1	Forward	GACTCAGAAGTCCCCGTCCC	61.61	65.00	NM_010512.5
	Reverse	GCATTTTCTGCTCCGTGGG	59.49	57.89	
Insr	Forward	ACCTTCTCTGATGAACGGCG	60.11	55.00	NC_000074.6
	Reverse	CTGATATGGGATCCAGGGGG	58.71	60.00	
Glut-1	Forward	GTTAATCGCTTTGGCAGGCGG	62.78	57.14	NM_011400.3
	Reverse	AGCATCTCAAAGGACTTGCCC	60.62	52.38	
Glut-2	Forward	GTGCTGCTGGATAAATTCGCC	60.27	52.38	NM_031197.2
	Reverse	TCAGCAACCATGAACCAAGGG	60.82	60.82	
Il-1β	Forward	TCTTTGAAGTTGACGGACCCC	60.20	52.38	NC_000068.7
	Reverse	CTTGTTGATGTGCTGCTGCG	60.73	55.00	
Nf-κb	Forward	TTTCGACTACGCAGTGACGG	60.39	55.00	NM_008689.2
	Reverse	GCTAAGTGTAAGACACTGTCCC	58.41	50.00	
Nox-4	Forward	CCTGCTCATTTGGCTGTCCC	59.96	50.00	NM_015760.5
	Reverse	GCTTAAACACAATCCTAGGCCC	59.97	55.00	
Sod-1	Forward	CATCCACTTCGAGCAGAAGGC	61.34	57.14	NM_011434.1
	Reverse	GGTACAGCCTTGTGTATTGTCCC	61.18	52.17	
Sod-2	Forward	GAACAACAGGCCTTATTCCGC	61.32	60.00	NM_013671.3
	Reverse	GTGTATCTTTCAGTAACATTCTCCC	59.31	50.00	
Gpx-3	Forward	GCACTACAAGAAGAACTTGGGC	59.77	50.00	NM_001329860.1
	Reverse	TCGAACATACTTGAGACTGGGG	59.50	50.00	
Cat	Forward	TGGTATAAGACGCATCAGAAGCC	60.49	47.83	NC_000071.6
	Reverse	GGTACTCCTCACTGAACATGCG	60.99	54.55	
β-Gluc	Forward	TGGTATAAGACGCATCAGAAGCC	60.49	47.83	NC_000071.6
	Reverse	GGTACTCCTCACTGAACATGCG	60.99	54.55	

Supplemental Table 2. Plasma lipid and glucose levels in mice exposed to protein restriction.

	Cholesterol (mg/dl)	LDL (mg/dl)	Triglycerides (mg/dl)	Glucose (mg/dl)
7 days				
Normal diet	244.0±94.0	30.4±20.0	216.0±142.0	89.4±51.0
Protein restriction	182.7±43.0	15.2±10.0	217.3±128.4	72.0±46.33
14 days				
Normal diet	244.5±130.3	22.5±23.0	225.0±155.9	115.0±53.7
Protein restriction	252.2±86.2	25.8±12.5	255.5±111.3	95.5±40.0
30 days				
Normal diet	252.1±120.0	32.7±29.7	259.3±147.2	97.7±46.0
Protein restriction	158.3±19.5*	13.6±14.1*	177.1±74.6*	70.4±35.7

*p<0.05 compared with corresponding normal diet.
